# Achalasia: Insights into Diagnostic and Therapeutic Advances for an Ancient Disease

**DOI:** 10.5041/RMMJ.10361

**Published:** 2019-01-28

**Authors:** Amir Mari, Kalp Patel, Mahmud Mahamid, Tawfik Khoury, Marcella Pesce

**Affiliations:** 1 Gastroenterology Institute, Nazareth EMMS Hospital, Nazareth, Israel; 2 The Azrieli Faculty of Medicine, Bar-Ilan University, Safed, Israel; 3 GI Physiology Unit, University College London Hospital, London, United Kingdom

**Keywords:** Achalasia, esophagogastric outflow obstruction, high-resolution manometry, per oral endoscopic myotomy (POEM)

## Abstract

Achalasia is a chronic idiopathic disease characterized by the absence of esophageal body peristalsis and by defective lower esophageal sphincter (LES) relaxation. The incidence rate ranges from 1.07 to up to 2.8 new cases per year per 100,000 population. Presenting symptoms include dysphagia, regurgitation, vomiting, and weight loss. The diagnosis of achalasia has undergone a revolution in the last decade due to the advent of high-resolution manometry (HRM) and the consequent development of the Chicago Classification. Recent progress has allowed achalasia to be more precisely diagnosed and to be categorized into three subtypes, based on the prevalent manometric features of the esophageal peristalsis. Treatment options are pharmacotherapy, endoscopic management (Botox injection or pneumatic dilation), and surgery, e.g. laparoscopic Heller myotomy (LHM). More recently, a new endoscopic technique, per oral endoscopic myotomy (POEM), has developed as a less invasive approach alternative to the traditional LHM. Since the first POEM procedure was performed in 2008, increasing evidence is accumulating regarding its efficacy and safety profiles. Currently, POEM is being introduced as a reasonable therapeutic option, though randomized controlled trails are still lacking. The current review sheds light onto the diagnosis and management of achalasia, with special focus on the recent advances of HRM and POEM.

## INTRODUCTION

**Figure 1:**
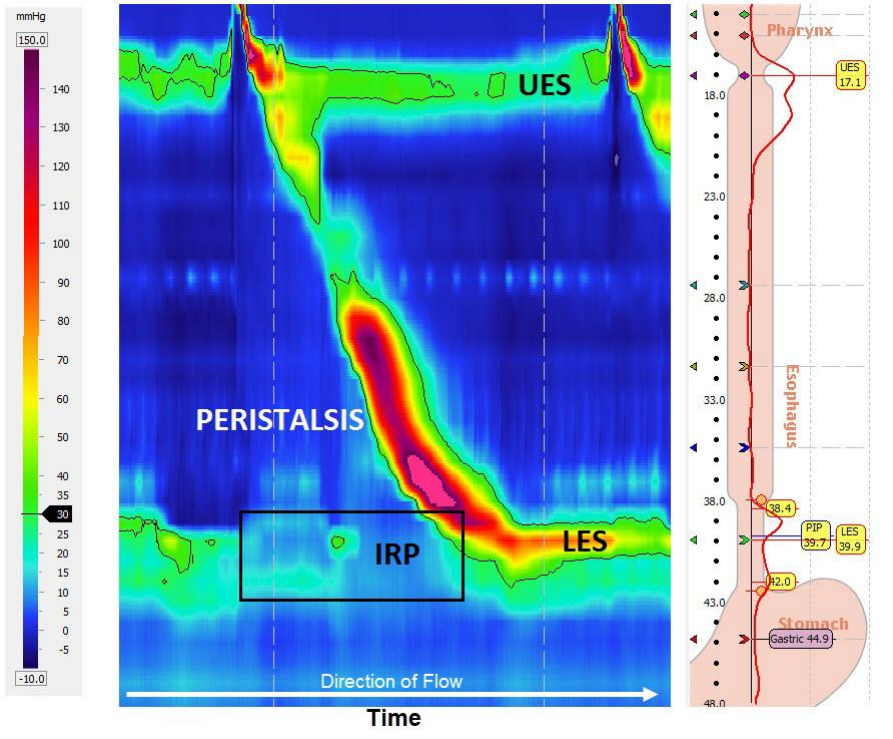
Example of High-resolution Manometry with Esophageal Pressure Topography Single swallow initiated at the upper esophageal sphincter (UES) showing normal esophageal body peristalsis and normal lower esophageal sphincter (LES) relaxation; LES relaxation is measured over a 10 second period as indicated by the black box and calculation of the integrated relaxation pressure (IRP).

The term “achalasia” originates from the Greek word *a-khalasis*, meaning lack of relaxation. It is a neurodegenerative disorder characterized by the absence of esophageal body peristalsis and by defective lower esophageal sphincter (LES) relaxation. The precise pathogenesis of this condition is poorly understood so far. Nonetheless, recent evidence suggests a possible role of an autoimmune reaction triggered by a viral infection that leads to an inflammatory process and consequent disruption of inhibitory neurons within the myenteric plexus, releasing nitric oxide.^1^,^2^ Achalasia is a rare disease with incidence rate of 1.63/ 100,000 population, and prevalence of 10/100.000 population,^3^ it is generally diagnosed between the ages of 30 and 60 years, and both genders appear to be equally affected.^3^ Its presenting symptoms are classically dysphagia, regurgitation of undigested food, vomiting, and weight loss. Less typical symptoms are heartburn, chest pain, cough, and choking. The diagnosis of achalasia is usually delayed for many years. The advent of high-resolution manometry (HRM) has largely replaced the traditional conventional manometry. Pressure recording is done by using a catheter with multiple closely spaced pressure sensors that traverses the esophagus and passes through the LES. This permits a colorful topographic presentation of esophageal body peristalsis as well as optimal localization of the LES (Figure 1). This has led to dividing achalasia into three distinct subtypes with different presentation, prognosis, and probably treatment response.^4^–^6^ Additionally, the presence or absence and size of a hiatal hernia can be assessed with HRM, with a higher sensitivity than with endoscopy or radiography alone.^7^

## DIAGNOSTIC APPROACH

The initial diagnostic step to the patient presenting with dysphagia is to rule out any mechanical or anatomic obstruction in the esophagus or at the esophagogastric junction (EGJ). Therefore, endoscopy is necessary to exclude tumors, inflammation, strictures, and other possible causes.^8^ Nearly half of achalasia patients will have suggestive findings of the disease during endoscopy, such as dilated esophagus, food and fluid contents, and difficulty with passing the endoscope through the EGJ.^9^ Barium-swallow serves as a complementary test and may show some morphological features suggestive of achalasia such as esophageal dilation and tapered distal esophagus. Timed barium-swallow (TBS) is an objective method to evaluate esophageal emptying by measuring the height of the fluid column over 5 minutes. Timed barium-swallow often represents a first-line diagnostic tool in patients complaining of dysphagia since it is inexpensive, non-invasive, and does not require special technology or expertise.^10^

**Figure 2:**
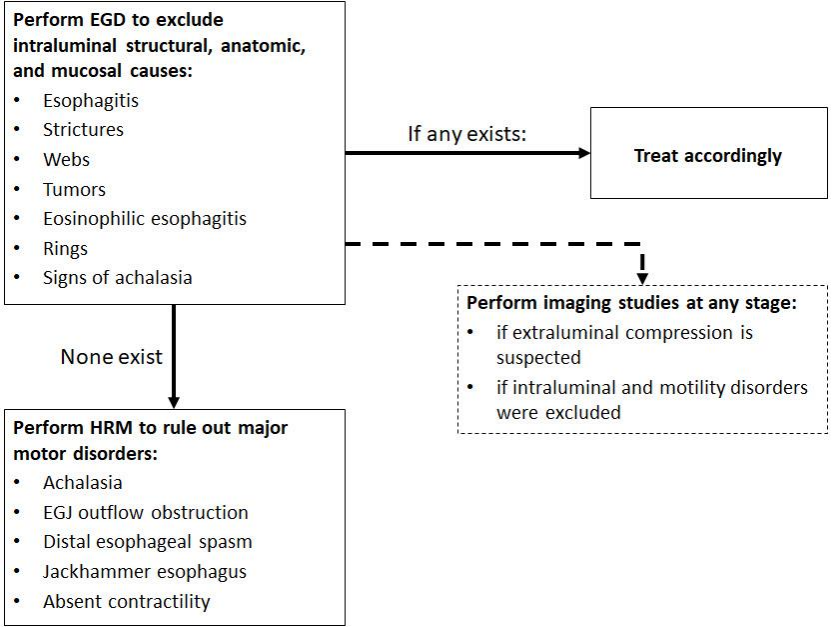
Diagnostic Algorithm in Patients with Symptoms of Esophageal Dysphagia EGD, esophago-gastro-duodenoscopy; EGJ, esophagogastric junction; HRM, high-resolution manometry.

Once mechanical or anatomical etiologies for the patient’s symptoms are excluded, HRM is the gold-standard investigation to be performed in order to search mainly for achalasia or EGJ outflow obstructtion as well as for other major peristaltic disorders (distal esophageal spasm, jackhammer esophagus, or absent contractility). An integrated diagnostic algorithm summarizing the stepwise approach to patients complaining of esophageal dysphagia is depicted in Figure 2.

## RECENT ADVANCES IN THE DIAGNOSIS OF DYSPHAGIA: HIGH-RESOLUTION MANOMETRY

High-resolution manometry is the gold standard modality in investigating patients complaining of dysphagia, diagnosing achalasia, and defining its subtype, and has largely replaced the traditional conventional manometry, used for the last 60 years.^11^ The HRM catheter contains up to 36 pressure sensors distributed closely (1 cm distance from each other) throughout its length. The catheter is introduced through the nares, traverses the esophagus, and passes through the EGJ. Each sensor records pressures during the study, and data are transferred to software. The software utilizes this information to create a visually instinctive topographic spatiotemporal plot of esophageal peristalsis and LES function, where changes in pressure are represented as color variations over a time axis (Figure 1). In 1991 Silny described the utility of intraluminal impedance to assess bolus propagation through the gastrointestinal tract.^12^ The system is based on measurement of electrical impedance (resistance to electrical current conduction), between several electrodes spanning an intraluminal catheter. Intraluminal air has high impedance, whereas liquid has low impedance. The addition of impedance function during HRM study has enabled an objective assessment of bolus movement through the esophagus, which reflects esophageal emptying.

There are several advantages of HRM compared to the old conventional manometry, including: better localization of the LES, shorter study time, less intra-observer and inter-observer variation of study analysis, and, lastly, optimal assessment of esophageal body peristalsis with the ability of detecting even minor peristaltic defects.^11^–^13^

The development of HRM has allowed the subsequent development of the Chicago Classification, currently at its third iteration, and the subsequent objective classification of esophageal motor disorders, thus improving standardization in the diagnosis and the follow-up of these disorders.^14^ The Chicago Classification is a practical scheme for analyzing and interpreting HRM studies as well as classifying esophageal motility into major and minor disorders.^15^,^16^ The latest version of the Chicago Classification was finalized in Chicago, USA, during an International HRM Working Group.^16^ This classification was then endorsed by several international motility societies, as an algorithm standardizing the interpretation of HRM studies.

**Figure 3:**
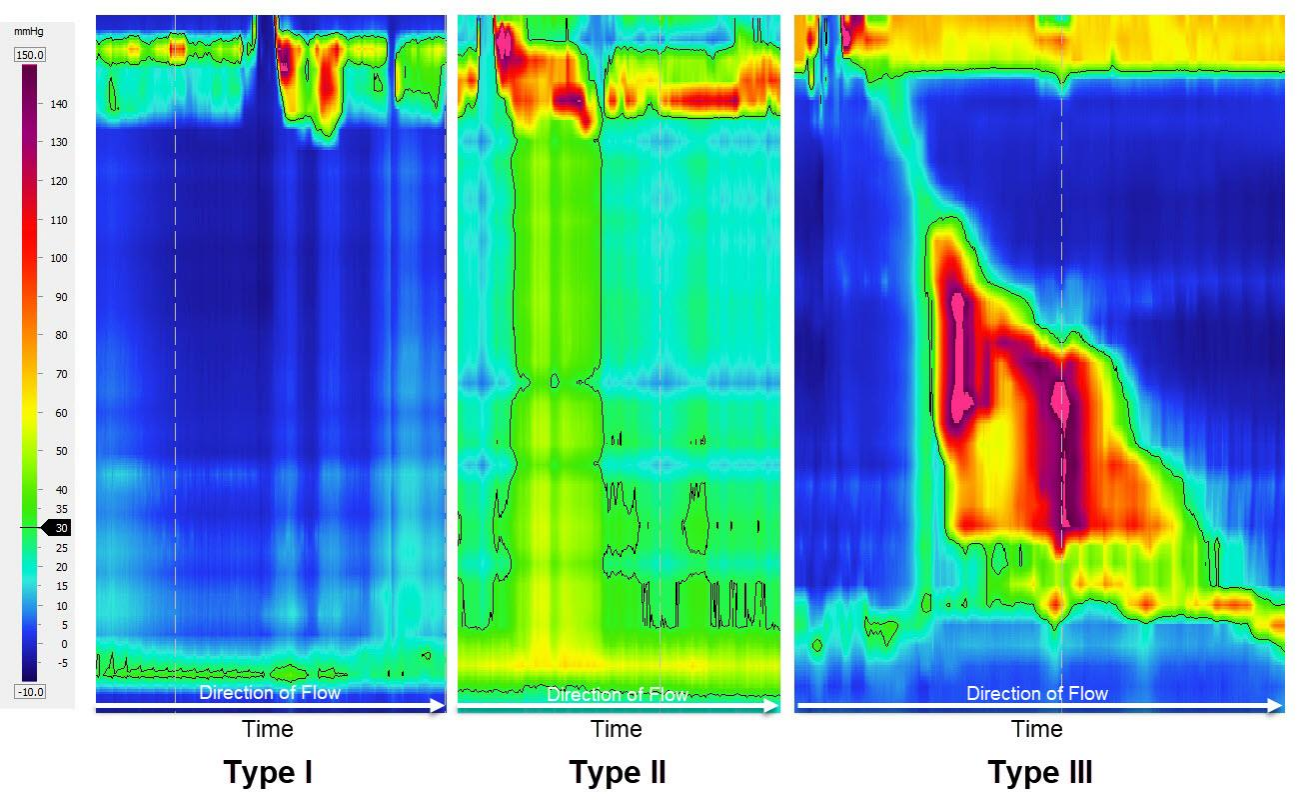
The Chicago Classification Achalasia Subtypes Achalasia type I is characterized by the total absence of peristalsis for all swallows. In type II the peristalsis is replaced by pan-esophageal pressurizations. Type III is characterized by the presence of premature spastic contraction.

The Chicago Classification subdivides achalasia into three subtypes (Figure 3). The presence of impaired EGJ relaxation is the common denominator of all subtypes with an increased integrated relaxation pressure (IRP), defined as the lowest average relaxation pressure within 10 seconds of LES relaxation window. An increased IRP (>15 mmHg) defines outflow obstruction and resistance to flow at the level of the EGJ.^16^ Achalasia type I is characterized by the total absence of peristalsis for all swallows. In type II the peristalsis is replaced by pan-esophageal pressurizations throughout the tubular esophagus, whilst type III is characterized by the presence of premature spastic contractions.^15^–^17^ This subdivision of achalasia has led to improved understanding of the different clinical presentations, prognosis, and also impacts on the therapeutic choices, allowing a tailored therapeutic strategy.^17^,^18^ One of the major observations with the classification of achalasia phenotypes was that treatment outcomes were dependent on phenotype, with outcomes being best in type II, intermediate for type I, and worst in type III.^19^ Rohof and colleagues have shown in a randomized controlled trial that the best treatment outcomes were observed in type I achalasia patients (more than 95% good treatment outcomes for balloon dilation and Hiller myotomy), while the least successful treatment outcomes were observed in type III achalasia patients (40% and 84% good treatment outcomes for balloon dilation and Hiller myotomy, respectively).^20^

**Figure 4:**
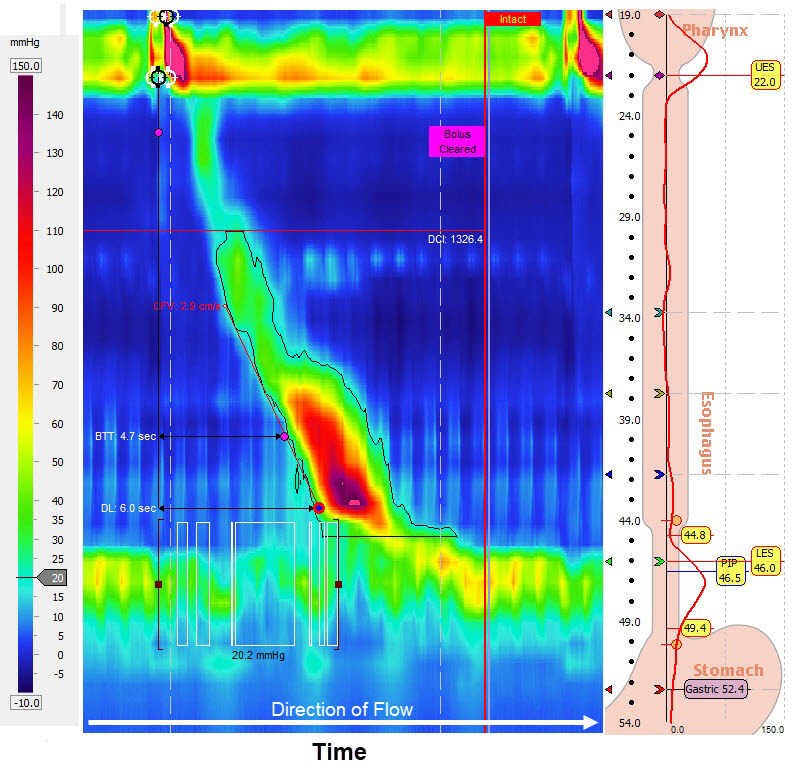
Esophagogastric Outflow Obstruction (EGOO) Shown is the outflow obstruction at the level of the esophagogastric junction (IRP>15 mmHg), accompanied by normal esophageal body peristalsis. BTT, bolus transit time; CFV, contraction front velocity; DL, distal latency; DCI, distal contractile integrity; PIP, pressure inversion point.

Additionally, the Chicago Classification has introduced a new entity called functional or idiopathic EGJ outflow obstruction—historically known as “variant achalasia.” This entity is now more commonly diagnosed due to the advent of HRM and is characterized by the evidence of outflow obstruction at the level of the EGJ, accompanied by normal esophageal body peristalsis (Figure 4).^16^ The diagnosis of EGJ outflow obstruction demands a careful endoscopic and imaging assessment in order to exclude misrecognized mucosal or anatomical pathologies, mainly submucosal tumors, previously known as pseudo-achalasia.^21^ Opiate use has been linked with esophagogastric outflow obstruction, hyperspasticity, and other motor disorders.^22^–^24^ Hence, it is also important to address that properly during history taking. Some investigators have suggested that functional EGJ outflow obstruction represents an early achalasia.^16^ Nevertheless, although EGJ outflow obstruction is being reported more frequently than achalasia, challenges exist in understanding its etiology, clinical significance, natural history, and appropriate therapy, and more research is warranted to better elucidate these uncertainties.

## TREATMENT OPTIONS

The main aim of achalasia treatment is to reduce patients’ symptoms and to improve their quality of life. Achalasia is an irreversible disease, and the restoration of esophageal peristalsis is unreliable. Therefore, the ultimate goal of therapy is to relieve the obstruction at the level of the EGJ by either administering drugs able of inducing LES relaxation (botulinum toxin injection) or by mechanically disrupting sphincter integrity via endoscopic (pneumatic dilatation or per oral endoscopic myotomy [POEM]) or surgical techniques (laparoscopic Heller myotomy [LHM]). The decision regarding the optimal treatment strategy largely depends on the patient’s symptoms, comorbidities, age, achalasia type, preference, and the center expertise.^25^

The pharmacological treatment of achalasia includes administration of several muscle-relaxant drugs such as nitric oxide, calcium channel blockers, or sildenafil. However, pharmacological agents have generally limited efficacy, temporary effect, and are associated with adverse events such as headache, edema, and dizziness.^26^ Thus, the use of pharmacological agents is limited to specific clinical situations such as a bridge therapy before more invasive procedures or when the patient is not candidate to invasive therapies.

Botulinum toxin injection at the EGJ has been used for achalasia treatment since 1994.^27^ The main effect of botulinum toxin is to block the release of acetylcholine which ultimately causes the temporary loss of muscle contraction. The main limitation of this treatment is its temporary efficacy, with a drop in symptom relief from 78% at one month to less than 40% after one year.^28^ Hence, botulinum toxin injection use is also limited to specific clinical situations such as a bridge therapy before an invasive procedure or for frail patients not candidates for more definitive therapies.^29^

Pneumatic balloon dilation is an effective and safe treatment for achalasia.^29^ During the procedure, an inflatable balloon is passed through the esophagus under fluoroscopic guidance and placed within the LES, and then the balloon inflated up to 10–15 pounds per square inch (PSI) and maintained for 60 seconds, leading to muscle disruption in the LES. The graded balloon dilation approach with increasing balloon diameters (30 mm, 35 mm, 40 mm) has been proven to be more efficient and safer, leading to fewer esophageal perforations.^30^ In most centers, pneumatic dilation is performed in two treatments with 30 mm and 35 mm balloon dilatations performed 2 to 4 weeks apart. Pneumatic balloon dilation is an efficient and long-lasting treatment, with a success rate of 86% after 2 years and 85% after 5 years.^30^,^31^ The long-term outcomes following pneumatic balloon dilation are comparable to those of LHM after 2 and 5 years.^32^ The main complications of pneumatic balloon dilation are gastro-esophageal reflux disease occurring in 15%–35% of patients, which is generally well controlled by anti-acid medications.^31^ The reported risk of esophageal perforation ranges from 2% to 4%, and the risk increases with balloon size, limited performer experience, and among men.^30^,^31^ Conservative management of esophageal perforations is satisfactory in most cases, with good short- and long-term prognosis.^33^

Heller myotomy has been performed for achalasia treatment for more than 100 years. The laparoscopic approach is the currently preferred one. During the surgery, a dissection of the anterior muscle fibers is performed. In order to prevent post-surgical gastro-esophageal acid reflux, a fundoplication is generally done after myotomy. Partial fundoplication, rather than a complete one, is the preferred approach in order to avoid dysphagia.^34^ So far, two types of partial wrap are commonly performed in achalasia patients after Heller myotomy: the posterior 270° fundoplication (Toupet) and the anterior 180° fundoplication (Dor).^35^ The success rate measured by symptom improvement scores following LHM is estimated to be 85% after 5 years.^36^ The surgery is safe with very low mortality rate, less than 1/1000.

## NOVEL ADVANCES IN THERAPY: PER ORAL ENDOSCOPIC MYOTOMY (POEM)

Inoue performed the first POEM in humans in 2008.^37^ The procedure’s objective is to perform LES myotomy during an endoscopic procedure. This is achieved by creating a submucosal tunnel accessed through a mucosal orifice within the esophageal wall, passing through the tunnel into the EGJ region, and then performing myotomy of the circular muscles mainly with an endoscopic knife.^38^ Since the first report published in 2010, numerous case series from all over the world have been published, contributing to the growing knowledge about POEM. Barbieri et al. published a meta-analysis in 2015 of 551 patients, showing a pooled success rate of 93%, though the follow-up period was very diverse and ranged from 3 months to 3 years.^39^ The major adverse events of POEM include: hydrothorax, late bleeding, pneumothorax, and intensive care unit admission. The major adverse events rate is 3.3% as shown in a retrospective study of 1680 Chinese patients.^40^ Analysis of three studies revealed comparable safety and efficacy profiles of POEM in comparison with balloon dilation and LHM, with a main advantage of POEM with shorter hospital stay and recovery time.^41^ Furthermore, it has been hypothesized that POEM could offer a more efficient symptomatic relief for type III achalasia patients in comparison with surgery.^42^ So far, no randomized controlled trials comparing POEM to balloon dilation or Heller myotomy have been performed. The indications of POEM are expanding to include major motility disorders other than achalasia, such as diffuse esophageal spasm and jackhammer esophagus refractory to conservative therapies. Khashab and colleagues showed a 93% clinical success rate of POEM after a 8-month follow-up period in these patients.^43^

To summarize, achalasia is a chronic motor disorder with unknown etiology. The advent of HRM in the last decade has permitted to improve our understanding of esophageal peristalsis and to consequently subclassify achalasia into three groups with different prognoses, providing a tailored therapeutic approach. The introduction of HRM alongside the advent of the POEM procedure, a novel therapeutic option for achalasia, has radically changed the approach to the disorder. The safety and efficacy of POEM have been shown to be comparable to classical techniques such as balloon dilation and surgery. Although these results are encouraging, randomized controlled trials are eagerly awaited to better define the long-term follow-up outcomes of POEM.

## Abbreviations

EGJ: esophagogastric junction
HRM: high-resolution manometry
LHM: laparoscopic Hiller myotomy
POEM: per oral endoscopic myotomy
LES: lower esophageal sphincter
TBS: timed barium-swallow.
